# 683. Understanding *Clostridioides difficile* toxinB gene conservation through surveillance of public data

**DOI:** 10.1093/ofid/ofad500.745

**Published:** 2023-11-27

**Authors:** Kelly Mulholland, Victoria Godfrey, Ann Marie Stanley, Tyler Brady, Bret R Sellman, Christine Tkaczyk, Vancheswaran Gopalakrishnan

**Affiliations:** AstraZeneca, Gaithersburg, Maryland; AstraZeneca, Gaithersburg, Maryland; Translational Medicine, Vaccines & Immune Therapies, BioPharmaceuticals R&D, AstraZeneca, Gaithersburg, MD, USA, Gaithersburg, Maryland; AstraZeneca, Gaithersburg, Maryland; AstraZeneca, Gaithersburg, Maryland; AstraZeneca BioPharmaceuticals R&D, Gaithersburg, Maryland; AstraZeneca, Gaithersburg, Maryland

## Abstract

**Background:**

*Clostridioides difficile* infection (CDI) is the leading cause of antibiotic and healthcare-associated infective diarrhea. It is linked to 365,000 infections and causes approximately 20,000 deaths annually in the US. CDI is mediated by the action of toxin B (TcdB) in humans. Monoclonal antibodies (mAbs) that neutralize TcdB function have shown protection against CDI in preclinical models and more recently in the clinic. Several regions on the TcdB gene have been targeted by therapeutic mAbs including the glucosyltransferase (GTD) and the C-terminal combined repetitive oligopeptides (CROPS) domains. We have developed AZD5148, an anti-TcdB mAb targeting a region within the GTD. To understand its strain coverage, we examined sequence variations of GTD including the AZD5148 epitope in contemporary *C. difficile* strains utilizing a public data repository.

**Methods:**

8,085 assembled *C. difficile* genomes collected between 2015-2022 were obtained from Enterobase. These were annotated with Prokka to identify the TcdB gene coding sequence, and subsequently aligned to the R20291 strain reference. Phylogeny was determined with ParSnp and visualized using iTol. From the aligned sequences, variants were identified using an in-house tool (RADAR).

**Results:**

A majority of the *C. difficile* genomes deposited were from North America, specifically the United States. Ribotype (RT) information was available for 9.6% of the genomes with RT-078 being the most common. Phylogenetic analysis did not reveal any patterns by geography or collection year, though clades tended to be associated with ribotypes. Degree of conservation was calculated at each TcdB genomic residue and was evaluated both regionally and temporally. We found GTD to be highly conserved in US isolates as well as globally. Additionally, among contact residues for AZD5148, any variations observed were single, and were seen for positions 323 (Y-H), 329 (G-E), 349 (V-A), and 350 (L-I).

**Conclusion:**

These findings have important implications for drug development including for AZD5148. Specifically, our findings reveal that the GTD domain of the TcdB gene has lower variance globally over the last 7 years making it a promising target for a *C. difficile* TcdB neutralizing mAb.

**Disclosures:**

**Kelly Mulholland, PhD**, AstraZeneca: Employee|AstraZeneca: Stocks/Bonds **Victoria Godfrey, MS**, AstraZeneca: Employee|AstraZeneca: Stocks/Bonds **Ann Marie Stanley, PhD**, AstraZeneca: Employee|AstraZeneca: Stocks/Bonds **Tyler Brady, MS**, AstraZeneca: Employee|AstraZeneca: Stocks/Bonds **Bret R. Sellman, PhD**, AstraZeneca: Employee|AstraZeneca: Stocks/Bonds **Christine Tkaczyk, PhD**, AstraZeneca: Employee|AstraZeneca: Stocks/Bonds **Vancheswaran Gopalakrishnan, PhD**, AstraZeneca: Employee|AstraZeneca: Stocks/Bonds

